# Ferroptosis-associated changes in transfusion-related acute lung injury in Sprague Dawley rats

**DOI:** 10.1515/med-2026-1418

**Published:** 2026-05-11

**Authors:** Chunli Cai, Chunqian Cai, Yaorong Lei, Huayu Chen, Qiao Xu, Jing He

**Affiliations:** First Teaching Hospital of Tianjin University of Traditional Chinese Medicine, Tianjin, China; National Clinical Research Center for Chinese Medicine, Tianjin, China; Laboratory Department, First Teaching Hospital of Tianjin University of Traditional Chinese Medicine, Tianjin, China; Acupuncture and Moxibustion Department, First Teaching Hospital of Tianjin University of Traditional Chinese Medicine, Tianjin, China; Medical Records Department, First Teaching Hospital of Tianjin University of Traditional Chinese Medicine, Tianjin, China

**Keywords:** transfusion-related acute lung injury, ferroptosis, biomarker

## Abstract

**Objectives:**

Transfusion-related acute lung injury (TRALI) is a severe transfusion complication, and oxidative stress has been implicated in its pathophysiology. Whether ferroptosis-associated changes are present in TRALI remains unclear.

**Methods:**

A two-hit TRALI-like model was established in Sprague Dawley (SD) rats. Animals were randomized into five groups: Control; lipopolysaccharide (LPS) (intraperitoneal, i.p., 2 mg/kg); Saline (i.p. LPS + saline); TRALI-like (i.p. LPS + human plasma); and LPS (intravenous, i.v., 5 mg/kg, positive control). Six hours after the second hit, the right lung lobe was collected for wet-to-dry (W/D) ratio assessment and haematoxylin and eosin (H&E) histology. Lung tissue glutathione (GSH), glutathione peroxidase 4 (GPX4), malondialdehyde (MDA), and prostaglandin-endoperoxide synthase 2 (PTGS2) protein expression were measured.

**Results:**

The TRALI-like group exhibited a higher W/D ratio and more severe histopathological changes than the LPS (i.p.) and Saline groups, indicating an additive effect of plasma transfusion within the two-hit framework. Increased lipid peroxidation and altered antioxidant defense were observed in the TRALI-like and LPS (i.v.) groups.

**Conclusions:**

Ferroptosis-associated alterations are present in this TRALI-like rat model and may be associated with lung injury. However, causal involvement cannot be concluded based solely on biomarker-based evidence.

## Introduction

Transfusion-related acute lung injury (TRALI) was first characterised in 1985, marking a critical advancement in understanding transfusion complications [1]. Clinically, TRALI presents within 6 h post-transfusion, manifesting as dyspnoea, fever, hypotension or hypertension, hypoxia and bilateral symmetric pulmonary oedema [2]. Autopsy findings in patients with TRALI commonly reveal pulmonary oedema, diffuse alveolar damage, hyaline membrane formation and significant granulocyte infiltration within the alveoli. Despite improvements in understanding TRALI’s pathophysiology, no specific therapies are available [3]. This highlights the pressing need for a deeper understanding of the underlying mechanisms involved in TRALI, which could pave the way for new diagnostic, therapeutic and preventive strategies. Notably, in preclinical settings where human plasma is administered to rodents, cross-species (xenogeneic) transfusion can elicit nonspecific pulmonary responses, including complement activation and fibrinogen-related effects, which may confound attribution of observed injury to classical TRALI mechanisms alone. Therefore, we conservatively refer to our experimental system as a “TRALI-like” model.

Recent research has reinforced the importance of the two-hit hypothesis in TRALI pathogenesis [4]. The first hit involves initial priming of the pulmonary vasculature, often due to underlying clinical risk factors in the recipient, which triggers the activation and sequestration of neutrophils in the lungs [5]. In experimental TRALI-like settings, this susceptible “first-hit” state can be modelled by low-dose inflammatory priming, which promotes pulmonary leukocyte sequestration and endothelial activation, thereby lowering the threshold for subsequent transfusion-mediated injury [4]. The first hit is not exclusively related to neutrophil stimulation; other factors, such as endothelial cell injury, complement activation and platelet aggregation, also play vital roles in initiating the inflammatory response. For instance, recent studies have expanded our understanding of the complex interactions between immune cells, particularly macrophages and the endothelial lining in the lungs during TRALI [6], 7]. The second hit occurs when sensitised blood products, particularly those containing antibodies or bioactive molecules, are transfused, leading to the activation of these neutrophils and the onset of TRALI [8], [9], [10], [11], [12]. Accordingly, our TRALI-like model follows this framework by applying LPS priming as the first hit and human plasma transfusion as the second hit, enabling evaluation of transfusion-related amplification of lung injury on a pre-inflamed background [4].

Macrophages serve as key regulators of the pulmonary inflammatory response, rapidly proliferating after the second hit and causing alveolar epithelial damage by activating downstream inflammatory pathways, oxidative stress and endoplasmic reticulum stress [13], 14]. Recent studies have provided further evidence of the central role macrophages play in mediating TRALI pathogenesis by amplifying the immune response and contributing to tissue injury [15], 16].

Moreover, the involvement of oxidative stress in TRALI has been increasingly recognised. Reactive oxygen species (ROS) generated during the inflammatory response are key contributors to tissue damage. Semple et al. demonstrated that an imbalance between ROS and antioxidants is a critical feature in TRALI, leading to oxidative damage and exacerbation of lung injury [17]. Importantly, excessive ROS production and impaired antioxidant defenses promote lipid peroxidation and disrupt cellular redox homeostasis, pathological processes that are also central to iron-dependent forms of regulated cell death [18]. The disruption of the redox balance in the lungs may further facilitate the inflammatory cascade, leading to the progression of TRALI.

Ferroptosis, a newly recognised form of regulated cell death driven by iron-dependent lipid peroxidation, may contribute substantially to the oxidative stress response associated with acute lung injury (ALI), including TRALI [19]. Unlike other forms of cell death, ferroptosis is characterised by lipid peroxidation accumulation, iron overload and glutathione (GSH) depletion, making it uniquely associated with oxidative damage and inflammation in pulmonary tissues [20]. Notably, these hallmark features of ferroptosis – oxidative stress, lipid peroxidation, and redox imbalance – closely overlap with the pathological processes known to characterise TRALI [21]. Although extensively studied in other diseases, such as cancer and acute kidney injury, ferroptosis may also play a role in ALI, which shares pathophysiological similarities with TRALI, such as oxidative stress and inflammatory responses [22]. For example, studies on ALI have shown that ferroptosis contributes to alveolar epithelial cell damage and exacerbates pulmonary inflammation, which aligns with the known features of TRALI [23]. Nevertheless, evidence from endotoxin- or sepsis-driven ALI models cannot be directly extrapolated to transfusion-triggered TRALI without testing within an appropriate two-hit experimental context.

Several studies have implicated ferroptosis in the development of ALI and sepsis-induced pulmonary damage, conditions which share pathophysiological mechanisms with TRALI, including neutrophil activation, oxidative stress, and endothelial dysfunction. For instance, Yin et al. and Pan et al. demonstrated that LPS-induced ALI in mice is associated with increased iron accumulation, lipid peroxidation, and decreased GPX4 expression-hallmarks of ferroptosis [24], 25]. These findings suggest that ferroptosis may represent a converging downstream pathway through which inflammatory injury and oxidative stress culminate in lung tissue damage, a concept that is highly relevant to TRALI pathogenesis [26], 27]. Furthermore, ferroptosis is a pharmacologically targetable process, and inhibitors such as ferrostatin-1 or liproxstatin-1 have been shown to alleviate lung injury in preclinical models. Therefore, investigating ferroptosis in TRALI is not only mechanistically relevant, but may also open a novel therapeutic avenue for transfusion-related lung injury.

Dixon et al. defined ferroptosis in 2012 as a process different from apoptosis, necrosis and autophagy [28]. However, its involvement in TRALI has yet to be explored in a transfusion-triggered, two-hit TRALI-like experimental framework. Given the recognised role of oxidative stress and lipid peroxidation in TRALI pathophysiology, we postulated that ferroptosis-associated alterations may be detectable in a TRALI-like setting and could be related to the severity of lung injury. Accordingly, we examined a panel of ferroptosis-associated biochemical and molecular readouts (including iron content, GSH, GPX4, MDA, and PTGS2) in a two-hit TRALI-like rat model.

Taken together, although oxidative stress is widely recognized as a key component of TRALI pathophysiology, whether ferroptosis-associated alterations are present in transfusion-triggered lung injury remains insufficiently explored, particularly within a two-hit experimental framework. Based on the overlap between TRALI-related oxidative stress and the molecular features of ferroptosis, we hypothesised that ferroptosis-associated changes may be detectable in a TRALI-like setting. Therefore, the primary research question of this study was whether ferroptosis-related biochemical and molecular alterations are present in a two-hit TRALI-like rat model induced by inflammatory priming followed by plasma transfusion.

## Materials and methods

### Rat model

A single unit of human whole blood was collected into a citrate phosphate dextrose (CPD) anticoagulant bag and processed into plasma by centrifugation (3,000 g, 10 min, 4 °C). The same batch of plasma was used for all rats in the TRALI-like group to ensure consistency. Plasma was clarified by passage through a 0.22 μm membrane filter to remove occasional cellular debris and microaggregates for procedural consistency and to minimise catheter occlusion during tail-vein infusion; this step was not intended to leukoreduce plasma or to modify the second-hit stimulus itself. The plasma was then aliquoted and stored at −80 °C. Prior to transfusion, plasma was thawed slowly at room temperature (20–25 °C) and administered within 30 min of thawing. No heat inactivation step was performed. In addition, the plasma was not subjected to heat inactivation or leukoreduction, in order to preserve labile bioactive components (e.g., immunoglobulins, complement activity, and other soluble inflammatory mediators) and to more closely reflect the clinical composition of transfused plasma. Heat inactivation may substantially alter plasma bioactivity (including complement and other proteins), potentially confounding interpretation of a TRALI-like response. Moreover, leukoreduction is not routinely applied to plasma in standard transfusion practice; therefore, we used unmodified plasma as the second-hit stimulus in this model.

A total of 25 male Sprague Dawley (SD) rats, aged 6–8 weeks and weighing 250–300 g, were randomly assigned into five groups (n=5 per group): Control group, LPS (i.p.) group (LPS-only; 2 mg/kg), Saline (volume control) group (i.p. LPS 2 mg/kg + saline), TRALI-like group (i.p. LPS 2 mg/kg + human plasma), and LPS (i.v.) group (positive control; 5 mg/kg). To establish a two-hit TRALI-like model, intraperitoneal LPS was used as the first hit to induce systemic inflammatory priming, and intravenous transfusion was used as the second hit to mimic transfusion-triggered lung injury. Human plasma transfusion was used as the intended second hit to model transfusion-related exposure following inflammatory priming, whereas LPS served to establish a susceptible inflammatory/oxidative background. The experimental timeline was as follows: (i) time 0, administration of LPS (2 mg/kg, i.p.) or an equal volume of sterile normal saline (Control); (ii) 2 h after priming, tail-vein blood removal (∼1 mL, approximately 10 % of blood volume) followed by equal-volume transfusion of human plasma (TRALI-like group) or sterile normal saline (Saline volume-control group) via the tail vein; (iii) 6 h after LPS administration, animals were euthanised and lung tissues were collected for downstream analyses. The LPS (i.p.) group received an intraperitoneal injection of lipopolysaccharide (LPS, 2 mg/kg, i.p.; Sigma-Aldrich, L2880) without any further intervention. The Saline (volume control) group received the same dose of LPS (2 mg/kg, i.p.), followed by blood removal (∼1 mL, equivalent to 10 % of blood volume) via the tail vein under sterile conditions. Two hours later, an equal volume of sterile normal saline was transfused via the tail vein. The TRALI-like group underwent the same LPS injection and blood removal procedure as the Saline group, but was transfused with human plasma instead of saline. The LPS (i.v.) group (positive control) received a high-dose intravenous injection of LPS (5 mg/kg, i.v.) to induce generalized ALI independent of plasma transfusion. The Control group received an intraperitoneal injection of an equal volume of sterile normal saline. The LPS (i.p.) group was designed to represent the first hit in the two-hit TRALI model, while the LPS (i.v.) group served as a positive control model of ALI independent of transfusion. The blood removal and equal-volume replacement step was included to standardise circulating volume across transfused groups and to ensure that observed effects were attributable to the transfused material rather than volume change.

At 6 h after LPS administration, the entire right lung (all lobes) was excised to measure the lung wet-to-dry (W/D) weight ratio as an indicator of pulmonary edema. The left lung was harvested for further analyses, including haematoxylin and eosin (H&E) staining, determination of iron content, GSH and MDA levels, and Western blot assays. To minimise sampling variability, all biochemical and protein analyses were performed using tissue collected from the left lung.

All SD rats were obtained from Beijing Vital River Laboratory Animal Technology Co., Ltd (Beijing, China) and housed under specific pathogen-free conditions (22 °C ± 2 °C, 50–60 % humidity, 12 h light/dark cycle) with free access to food and water.

### Analysis of iron content in lung

Lung tissues were removed and homogenised in an ice bath for 10 min for subsequent studies. Samples were processed step by step, heated in a boiling water bath for 5 min and cooled and centrifuged at 3,000 g for 10 min. Iron concentration in the rat lung was measured using an iron assay kit in accordance with the manufacturer’s protocol (Jiancheng Bioengineering Institute, China). In this method, under the action of an acidic solution and reductant, iron in ferritin is released from protein. After ferric iron is reduced to its ferrous form, it reacts with sulfadiazine and trimethoprim to form stable, coloured complexes. Within a certain range, the amount of iron is proportional to the colour intensity. Accordingly, the absorbance/optical density (OD) of the resulting coloured supernatant was measured at 520 nm as the final quantitative readout for iron determination (OD_520_).

### Glutathione assay

Lung tissues were homogenised on ice (1 g tissue in 9 mL NS). After centrifuging the homogenates at 2,000 g for 10 min, the supernatant was used for GSH assays. Total GSH levels were measured using the GSH assay kits (Jiancheng Bioengineering Institute, China) following the manufacturer’s instructions.

### Malondialdehyde assay

Lung tissues were homogenised. After centrifuging the homogenates at 3,000 rpm for 5 min at 4 °C, the MDA levels in rat lung tissues were analysed using an MDA assay kit (Jiancheng Bioengineering Institute, China). Malondialdehyde reacts with thiobarbituric acid under acidic conditions at a high temperature to form a stable chromophoric product, which is measurable at a wavelength of 532 nm. Absorbance measurements were performed using a UV-Vis spectrophotometer (model UV-1800, Shimadzu Corporation, Kyoto, Japan). The instrument was calibrated according to the manufacturer’s instructions, and each sample was measured in technical triplicate to ensure accuracy.

### Haematoxylin and eosin (H&E) staining

After immersion in 4 % paraformaldehyde for 24 h, lung tissues were transferred to 70 % ethanol for further preservation. The tissues were then embedded in paraffin blocks, sectioned at a thickness of 5 µm and stained with H&E. The stained sections were examined under a light microscope to assess the pathological changes in the lung tissues.

### Western blot

Lung tissue protein samples from the SD rats were lysed using cold cell lysis buffer and conducted to sodium dodecyl sulfate–polyacrylamide gel electrophoresis. Proteins in gels were transferred onto polyvinylidene difluoride membranes, followed by blocking at room temperature for 2 h using 1 × Blotto (blocking solution) and incubation with primary antibodies including anti-glyceraldehyde-3-phosphate dehydrogenase (GAPDH) antibody (Saier Biotechnology, China), anti-prostaglandin-endoperoxide synthase 2 (PTGS2) antibody (Saier Biotechnology, China) and anti-GPX4 (Sanying Wuhan, China). Primary antibody staining was conducted by incubating the membrane in Blotto containing the specific primary antibody at 4 °C overnight with gentle shaking. Afterwards, the horseradish peroxidase-conjugated antibody (HRP-conjugated anti-rabbit IgG) was incubated at room temperature for 1.5 h in Blotto. The chemiluminescence method using Western Lightning™ Chemiluminescence Reagent was used to detect protein expression, and the membrane was exposed to X-ray film for 1 min in a darkroom. The films were subsequently developed and fixed. Relative expression levels were calculated by analysing the brightness of the target protein bands using the LabWorks™ gel imaging and analysis system. The intensity of each target protein band was normalised to the corresponding GAPDH band intensity. The normalised values were then compared with the control group, which was set to a standard value of 1, and the results were presented as a bar graph.

For Western blot analysis, three animals per group (n=3) were randomly selected from each experimental group using a computer-generated list. This selection was performed prior to protein extraction and was not based on any observed phenotypic characteristics. Due to limited availability of lung tissue following completion of other biochemical and histological analyses, additional biological replicates could not be included. Therefore, Western blot results are presented as supportive molecular evidence and were interpreted with appropriate caution.

### Lung wet-to-dry weight ratio

Lung tissues were excised and weighed immediately. After the blood on the lung surface was rinsed off, tissues were oven dried at 60 °C for 72 h and reweighed. The lung W/D weight ratio, calculated by dividing the wet weight of the initial specimen by its dry weight, served as an indicator of lung oedema.

### Statistical analyses

All experiments were conducted independently with biological replicates as specified in each assay. The data were presented as mean ± standard deviation (SD). The normality of the data was assessed using the Shapiro–Wilk test and quantile–quantile (Q–Q) plot inspection. One-way analysis of variance (ANOVA) with Tukey’s honestly significant difference (HSD) post hoc test was used for multiple group comparisons (n=5 groups). A p-value <0.05 was considered statistically significant. All statistical analyses were conducted using IBM Statistical Package for the Social Sciences (SPSS) software, Version 28.

### Ethical approval

All animal experimental procedures were approved by the Institutional Animal Care and Use Committee and were conducted in accordance with the National Institutes of Health Guide for the Care and Use of Laboratory Animals and relevant institutional guidelines.

## Results

### A two-hit TRALI-like lung injury model was established

We successfully established a two-hit TRALI-like lung injury rat model using LPS priming followed by human plasma transfusion. In the TRALI-like group, rats received intraperitoneal LPS (2 mg/kg), underwent blood removal equivalent to 10 % of the circulating blood volume (≈1 mL), and were then transfused with an equal volume of human plasma 2 h later. This model reproduced TRALI-like pathological features (pulmonary oedema and inflammatory lung injury) within a two-hit experimental framework; however, it did not aim to fulfil all clinical TRALI diagnostic criteria.

To help distinguish LPS-driven injury from transfusion/volume-related effects, we included two additional control groups: an LPS (i.p.) group (LPS-only; 2 mg/kg, without transfusion) and a Saline group (volume control; i.p. LPS 2 mg/kg followed by equal-volume sterile saline infusion after blood removal). These groups were compared with the Control group and the LPS (i.v.) group (positive control; 5 mg/kg). The sample size for W/D ratio and histology was n=5 animals per group.

**Figure 1:**
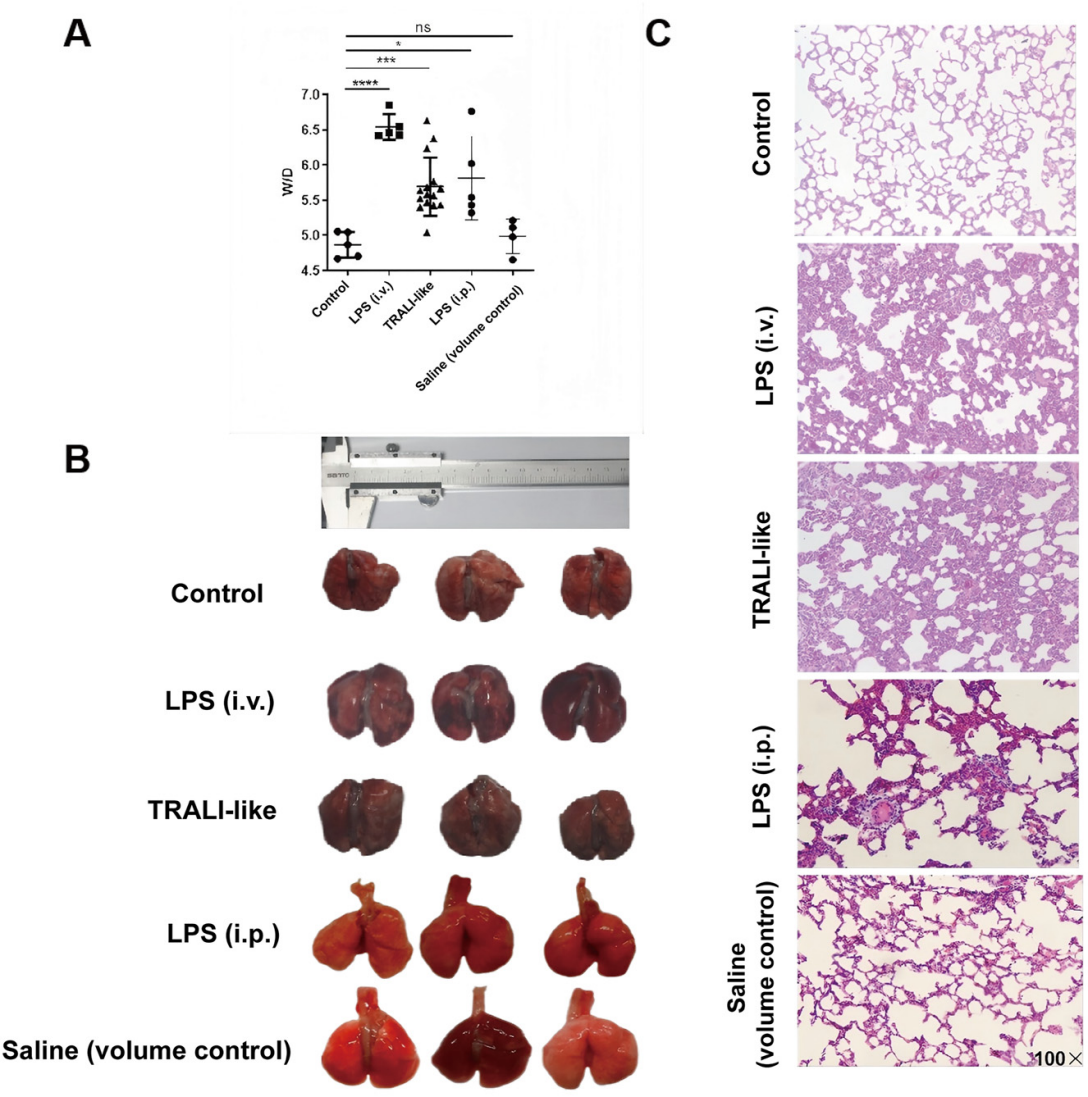
Establishment of the TRALI-like rat model and comparison of lung injury. (A) Lung wet-to-dry (W/D) weight ratio in the following groups: Control group, LPS (i.v.) group (positive control; 5 mg/kg), TRALI-like group (i.p. LPS 2 mg/kg + human plasma), LPS (i.p.) group (LPS-only; 2 mg/kg), and Saline (volume control) group. n=5 animals per group. (B) Representative macroscopic images of excised lung tissue from each group showing the extent of lung congestion and edema. n=5 animals per group. (C) Representative histopathological images of lung tissues stained with haematoxylin and eosin (H&E), demonstrating alveolar structure and infiltration in different groups (original magnification, ×100). n=5 animals per group. Data are presented as mean ± SD. *p<0.05, **p<0.01, ***p<0.001, ****p<0.0001. (LPS, lipopolysaccharide; TRALI, transfusion-related acute lung injury).

As shown in Figure 1A and B, the W/D ratio increased in LPS-exposed groups, and the TRALI-like group exhibited a comparable W/D ratio to the LPS (i.p.) group. The W/D ratio in the Saline (volume control) group was similar to that in the Control group, suggesting that equal-volume saline replacement did not further aggravate pulmonary oedema under these experimental conditions. The W/D ratio in the LPS (i.v.) group was the highest among all groups, and the corresponding statistical outputs (raw values, exact p values, and test statistics) are provided in Supplementary Table S1, rather than being described qualitatively.

Histological examination (Figure 1C) revealed more prominent inflammatory injury features in the TRALI-like group, including alveolar wall thickening, interstitial congestion, and increased inflammatory cell infiltration, compared to the LPS (i.p.) group. Together, these data suggest that human plasma transfusion after LPS priming may act as a second-hit stimulus that exacerbates lung pathology, whereas LPS priming alone appears sufficient to drive oedema formation as reflected by W/D ratio. Accordingly, this model is described as TRALI-like to reflect transfusion-triggered amplification of lung injury on an LPS-primed background.

### Ferroptosis-associated alterations in lung tissues

Ferroptosis is a type of regulated cell death characterised by GSH depletion, decreased GPX4 activity and diminished cellular antioxidant capacity, leading to increased lipid peroxidation. Elevated levels of ROS can trigger ferroptosis, particularly when there is abnormal iron accumulation.

To assess ferroptosis-associated changes in this experimental setting, pulmonary iron levels and the expression of prostaglandin-endoperoxide synthase 2 (PTGS2) and GPX4 were examined. Because PTGS2 is also an inflammation-responsive readout (particularly in LPS-driven injury), it was interpreted only as a supportive, non-specific indicator and not as standalone evidence of ferroptosis. Raw densitometry values and the corresponding statistical outputs for Western blot quantification are provided in Supplementary Table S2 (n=3 animals per group for Western blot). The BCA-derived protein concentration values used for sample normalization/loading are provided in Supplementary Table S3.

**Figure 2:**
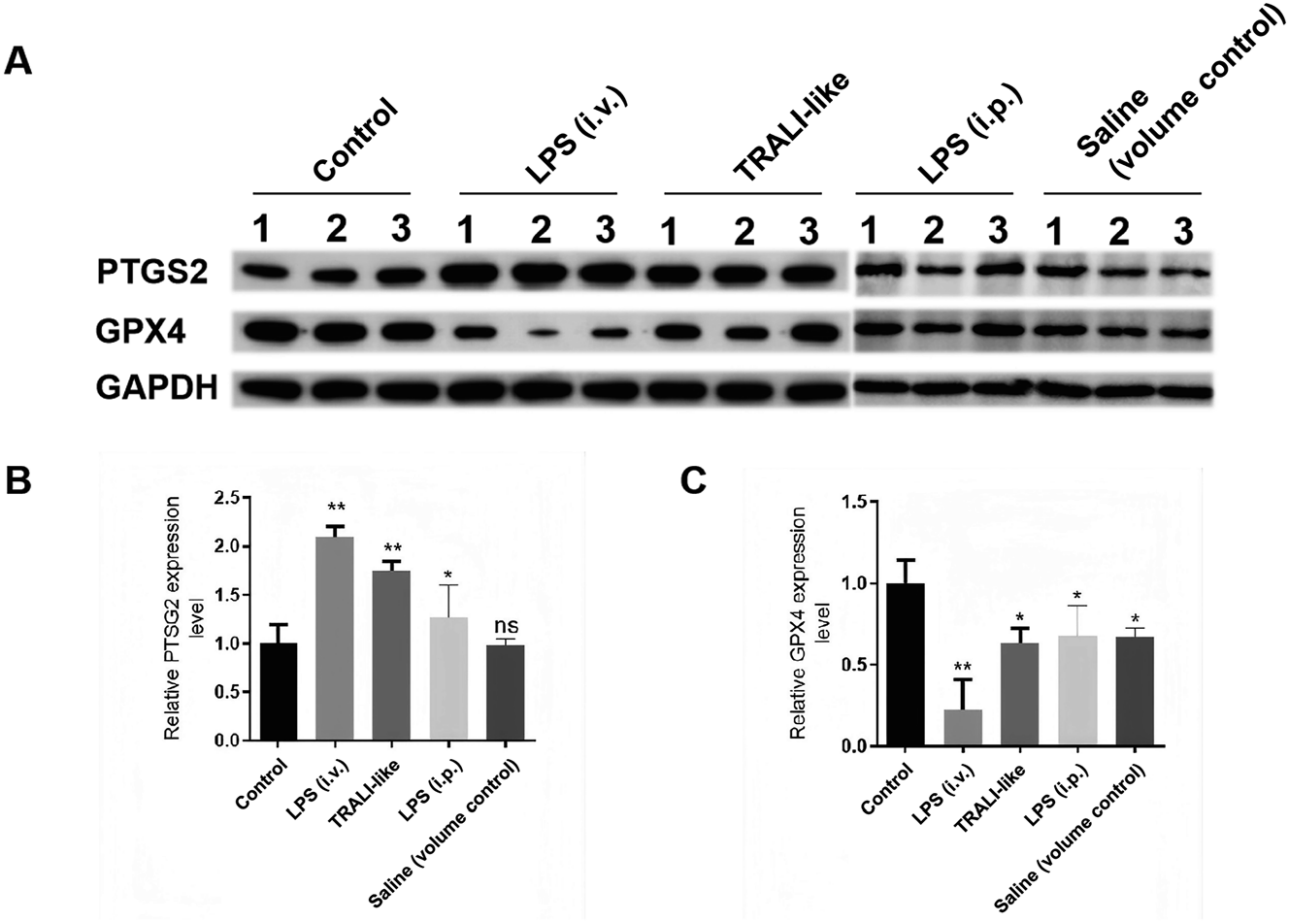
Protein expression of ferroptosis markers in each experimental group. (A) Western blot analysis of PTGS2 and GPX4 expression in lung tissues from the following groups: Control group, LPS (i.v.) group (positive control; 5 mg/kg), TRALI-like group (i.p. LPS 2 mg/kg + human plasma), LPS (i.p.) group (LPS-only; 2 mg/kg), and Saline (volume control) group. n=3 animals per group. (B) Quantification of PTGS2 protein levels relative to GAPDH across all groups. n=3 animals per group. Asterisks indicate statistical comparisons vs. the Control (normal) group. (C) Quantification of GPX4 protein levels relative to GAPDH across all groups. n=3 animals per group. Asterisks indicate statistical comparisons vs. the Control (normal) group. Data are presented as mean ± SD. *p<0.05, **p<0.01, ***p<0.001, ****p<0.0001 vs. the Control (normal) group. LPS, lipopolysaccharide; TRALI, transfusion-related acute lung injury; PTGS2, prostaglandin-endoperoxide synthase 2; GPX4, glutathione peroxidase 4.

As shown in Figure 2A and B, PTGS2 expression was increased in the TRALI-like group and also increased in the LPS (i.p.) group compared with the Control group. Importantly, PTGS2 did not show a clear TRALI-like–specific elevation beyond the LPS-only condition (Figure 2B), and the TRALI-like vs. LPS (i.p.) comparison did not reach statistical significance (Supplementary Table S2). PTGS2 expression in the Saline (volume control) group was comparable to that in the Control group. Therefore, under the present experimental conditions, the PTGS2 pattern is most consistent with an LPS-driven inflammatory/oxidative response rather than a plasma-specific ferroptosis signal. Accordingly, PTGS2 is presented as a supportive readout interpreted alongside GPX4 and biochemical oxidative stress indices, rather than as a discriminating ferroptosis marker.

### Oxidative stress and ferroptosis-associated biochemical profile

To further characterise oxidative stress–related changes associated with ferroptosis, lipid peroxidation and antioxidant status were evaluated by measuring malondialdehyde (MDA) and GSH levels, respectively.

As shown in Table 1, pulmonary iron content was higher in LPS-exposed groups (including the LPS (i.p.) group, Saline group, and TRALI-like group) than in the Control group. Exact p values for all pre-specified comparisons are reported numerically in Table 1 and Supplementary Table S4 (raw biochemical values and statistical outputs), rather than being described using qualitative “trend” language. Between-group separation among LPS-exposed conditions was limited, indicating that pulmonary iron accumulation was predominantly associated with LPS exposure under this model.

**Table 1:** Levels of GSH, iron concentration and MDA in lung tissues.

Indicator	Control group	LPS (i.v.) group	TRALI-like group	LPS (i.p.) group	Saline (volume control) group
GSH, μmol/g protein	31.3026 ± 2.0029	8.0959 ± 1.6529	16.0205 ± 2.1110	10.6423 ± 1.8745	9.3123 ± 1.5345
Iron concentration, μmol/g protein	10.3334 ± 1.9422	23.7643 ± 3.6024	16.3648 ± 1.2196	12.3984 ± 1.6545	14.5454 ± 1.2123
MDA, μmol/g protein	0.8324 ± 0.0668	1.6561 ± 0.1271	1.1060 ± 0.1557^a^	1.2762 ± 1.743	1.8432 ± 0.9433

Group definitions: Control, intraperitoneal saline; LPS (i.v.), LPS 5 mg/kg intravenous (positive control); TRALI-like, LPS 2 mg/kg intraperitoneal + human plasma transfusion (second hit); LPS (i.p.), LPS 2 mg/kg intraperitoneal only; Saline (volume control), LPS 2 mg/kg intraperitoneal + equal-volume saline transfusion. All measurements were performed at 6 h after the second hit/time-matched point. Data are presented as mean ± SD (n=5 per group). Statistical analysis was performed using one-way ANOVA followed by Tukey’s post hoc test. ^a^p<0.05 compared with the Control group.

Similarly, MDA levels were higher in LPS-treated groups relative to the Control group, and the TRALI-like group did not demonstrate a distinct increase beyond LPS-only conditions. GSH levels were reduced in LPS-treated groups. Any between-group differences among LPS-exposed conditions should therefore be interpreted cautiously and in accordance with the numerical statistical outputs provided (Table 1; Supplementary Table S4).

Western blot analysis demonstrated that GPX4 protein expression was reduced in LPS-exposed groups compared with the Control group (Figure 2A and C). Given the limited Western blot biological replicates (n=3 per group), these results should be interpreted as supportive and hypothesis-generating rather than definitive for group separation.

Taken together, the concurrent iron elevation, increased MDA, reduced antioxidant capacity, and reduced GPX4 expression indicate the presence of an oxidative stress–associated ferroptosis-related profile in this model. Because these alterations were observed across LPS-treated groups, they are most consistent with a shared downstream response to LPS-driven lung injury, with plasma transfusion potentially acting as a second-hit amplifier of overall pathology rather than a unique initiator of the measured ferroptosis-associated markers. To further explore whether injury severity aligned with ferroptosis/oxidative stress indices, we performed a simple correlation analysis between the lung W/D ratio and tissue iron, MDA, and GSH (and GPX4 densitometry where available). The correlation coefficients and exact p values are reported in Supplementary Table S5. Correlations involving GPX4 were interpreted as exploratory due to the limited Western blot biological replicates (n=3 per group).

## Discussion

In the present study, we found that ferroptosis-associated biochemical and molecular alterations were detectable in a two-hit TRALI-like rat model, characterised by inflammatory priming followed by plasma transfusion. This study successfully established a TRALI-like rat model and demonstrated the presence of ferroptosis-associated molecular alterations in this condition. The increased iron content in lung tissues, decreased GSH levels and reduced GPX4 expression observed in the TRALI-like rat model are consistent with the hallmark features of ferroptosis. Importantly, these alterations were observed in the context of a two-hit experimental design and were not exclusive to plasma transfusion alone, indicating that ferroptosis-associated changes may occur as part of a broader inflammatory and oxidative stress response in this TRALI-like setting. In this regard, the value of the present work is to place ferroptosis-associated alterations within a transfusion-triggered, two-hit TRALI-like framework, which differs conceptually and translationally from endotoxin-only ALI models. However, because these conclusions are derived from biomarker-based observations in the absence of functional or interventional modulation of ferroptosis, the present findings should be interpreted as associative and hypothesis-generating rather than causative. Specifically, the observed ferroptosis-associated changes likely reflect a shared downstream oxidative stress response in the TRALI-like setting, rather than evidence of a TRALI-specific ferroptotic mechanism. Importantly, by evaluating ferroptosis-associated alterations within a transfusion-triggered, two-hit TRALI-like framework, our study helps bridge evidence largely derived from general ALI settings (often endotoxin- or sepsis-driven) with transfusion-related lung injury, thereby improving the contextual relevance of ferroptosis-related hypotheses to TRALI-like conditions. Notably, species differences between rodents and humans may influence inflammatory and oxidative injury responses, and therefore direct clinical extrapolation from SD rat models should be made cautiously. Nevertheless, ferroptosis-associated features such as iron dysregulation, lipid peroxidation, and impaired antioxidant defense have been reported in human acute lung injury/ARDS contexts, supporting the biological plausibility that similar pathways may also be relevant to transfusion-associated lung injury in humans [29], 30].

Generally, the infusion of blood components containing antibodies against anti-homologous leukocyte antigens and anti-neutrophil antigens is considered an important factor in inducing TRALI [31], [32], [33], [34]. Transfusion-related ALI induced by neutrophil activation due to antibody interaction is referred to as antibody-mediated TRALI [35], [36], [37]. Nevertheless, not all TRALI cases are clinically associated with antibodies, and retrospective studies have confirmed that the accumulation of cellular mediators in blood donors and related changes during the storage of red blood cells and platelets are also associated with non-antibody-mediated TRALI [38], 39]. There is also controversy regarding the role of platelets in antibody-mediated TRALI. Some studies have suggested that platelets may contribute to the pathogenesis of TRALI by enhancing leukocyte activation, although this remains debated [40], 41]. Beyond neutrophils, macrophage activation has increasingly been recognised as a central component of TRALI pathogenesis, particularly in amplifying inflammatory signaling and oxidative stress within the pulmonary microenvironment [42].

Other forms of lung injury, particularly LPS-induced lung injury, have been widely studied and share common mechanisms with TRALI. A commonly used model for ALI is LPS-induced lung injury, in which inflammatory responses, oxidative stress and cellular apoptosis play central roles in the pathogenesis. As a bacterial endotoxin, LPS stimulates the immune system to release pro-inflammatory cytokines, leading to pulmonary inflammation, oedema and injury. Recent studies have shown that ferroptosis, triggered by oxidative stress and lipid peroxidation, also contributes to LPS-induced lung injury. Elevated lipid peroxidation and iron accumulation in lung tissues have been observed in experimental models of LPS-induced ALI, indicating that ferroptosis represents a common downstream consequence of inflammatory and oxidative stress, rather than a mechanism specific to plasma transfusion, and mirroring several ferroptosis-related features observed in the present TRALI-like model [43]. Consistently, inflammatory activation itself can upregulate genes that overlap with “ferroptosis marker panels”, reinforcing the need to distinguish ferroptosis-associated oxidative injury from non-specific inflammation-driven changes.

Sepsis, which is commonly associated with LPS exposure, is a serious clinical condition that induces systemic inflammation and multiple organ failure, including ALI [44]. Ferroptosis has been linked to sepsis-induced ALI, where elevated iron levels and lipid peroxidation exacerbate the lung injury [45]. These observations further support the concept that ferroptosis may act as a shared downstream effector pathway across different inflammatory lung injury contexts, including LPS-induced ALI and TRALI-like lung injury. Accordingly, ferroptosis may represent a pathophysiologically relevant but not disease-specific process, highlighting its potential value as a therapeutic target while underscoring the need for caution in attributing ferroptosis activation exclusively to plasma transfusion in TRALI.

Bridging this gap is significant for two reasons. First, most mechanistic and therapeutic insights into ferroptosis in lung injury have been developed in general ALI contexts (often driven by endotoxin or sepsis), whereas transfusion-triggered lung injury represents a distinct initiating exposure operating within a clinically relevant two-hit paradigm. Placing ferroptosis-associated readouts into a transfusion-triggered framework therefore reduces reliance on direct extrapolation from endotoxin-only ALI studies and provides a more appropriate experimental context for evaluating whether ferroptosis-related processes are detectable in TRALI-like injury. Second, our findings support the concept that ferroptosis-associated alterations may reflect a shared downstream oxidative injury program across heterogeneous ALI triggers, while also highlighting the need for cautious interpretation of inflammation-responsive markers when translating ferroptosis “signatures” into transfusion-triggered settings.

This perspective suggests several future research directions. Future studies should (i) strengthen mechanistic specificity by expanding ferroptosis-relevant assessments alongside standardized pathological scoring; (ii) incorporate additional control conditions (including plasma-only transfusion and plasma-processing controls) to better separate priming-dependent injury from transfusion-specific amplification; and (iii) test ferroptosis-targeted interventions within transfusion-relevant models to determine whether ferroptosis is merely an associated signature or a modifiable driver/amplifier of TRALI-like lung injury. In parallel, cell-type–resolved approaches may clarify whether ferroptosis-associated processes primarily involve alveolar epithelium, endothelium, macrophages, or neutrophils within the two-hit cascade.

With respect to treatment development, ferroptosis is pharmacologically targetable in principle, and if future interventional work demonstrates a causal contribution in transfusion-triggered lung injury, ferroptosis-targeted strategies may be explored as adjunctive approaches for TRALI-like injury and potentially for broader ALI phenotypes in which iron dysregulation and lipid peroxidation are prominent. However, because the present study is biomarker-based and does not include ferroptosis modulation, any therapeutic implications should be considered hypothesis-generating and require dedicated validation in transfusion-relevant experimental and clinically translatable settings. In parallel, translational efforts would benefit from studies directly examining ferroptosis-related signatures in human TRALI/ARDS biospecimens and from assessing whether comparable oxidative-iron-lipid peroxidation patterns correlate with clinical severity or outcomes [46], 47].

We observed that the W/D ratio in the intraperitoneal LPS (i.p.) group was increased compared with the Control group and was comparable to that in the TRALI-like group, indicating that intraperitoneal LPS alone is sufficient to induce pulmonary edema. However, histopathological examination revealed more pronounced alveolar wall disruption, interstitial congestion, and inflammatory infiltration in the TRALI-like group, suggesting that human plasma transfusion following LPS priming exerts an additive pathological effect within a classical two-hit framework. Notably, both the TRALI-like group and the LPS (i.p.) group exhibited comparable elevations in ferroptosis-associated biomarkers, including PTGS2 and MDA, indicating that these biochemical changes are not specific to plasma transfusion but rather reflect a shared LPS-driven inflammatory and oxidative stress response. Given that PTGS2 is a well-recognized inflammation-responsive gene, the similar PTGS2 elevation in the LPS-only condition suggests that PTGS2 upregulation in this model likely reflects nonspecific inflammatory activation rather than a TRALI-specific ferroptotic signature. The greater histological severity observed in the TRALI-like group therefore supports a qualitative amplification of lung injury severity, rather than a TRALI-specific or quantitatively distinct increase in ferroptosis marker expression. The TRALI-like group also received a lower-dose inflammatory stimulus (i.p. LPS 2 mg/kg) compared with the positive control group (i.v. LPS 5 mg/kg), yet still exhibited comparable or more complex ferroptosis-associated alterations, suggesting that plasma transfusion may function as a second-hit amplifier of pre-existing LPS-induced inflammatory and oxidative stress responses, rather than independently initiating it. In contrast, although the W/D ratio in the LPS + saline group was similar to that in the Control group, this observation should be interpreted cautiously, as no direct evidence supports a protective effect of saline beyond its role as a volume control. Taken together, these findings, supported by gross morphology and H&E staining, confirm the successful establishment of a TRALI-like rat model within a two-hit experimental framework. Nevertheless, because a plasma-only transfusion group was not included, the independent contribution of plasma transfusion cannot be definitively separated from LPS priming in the present study, and therefore the observed plasma-related effects should be interpreted in the context of LPS-dependent susceptibility, rather than as evidence of plasma-driven lung injury or TRALI-specific ferroptosis occurring independently of LPS priming. Accordingly, PTGS2 was considered a supportive, non-specific readout in the present study and was not treated as standalone evidence for ferroptosis. Taken together, these patterns support the interpretation that plasma transfusion primarily modulates or amplifies an existing LPS-driven oxidative and inflammatory milieu rather than uniquely initiating ferroptosis-associated changes.

To further investigate the underlying mechanism of injury, we assessed the expression of PTGS2, a marker associated with ferroptosis [48]. However, PTGS2 is not specific to ferroptosis and can be robustly induced by inflammatory stimulation (including LPS), which limits its interpretability as a discriminating ferroptosis marker in the current model. Therefore, PTGS2 was interpreted only in conjunction with oxidative stress–related indices (iron accumulation, lipid peroxidation, and antioxidant depletion), rather than as a definitive indicator of ferroptosis. GPX4, an antioxidant enzyme, reduces intracellular lipid hydroperoxides to alcohols with the assistance of GSH, thereby maintaining cellular redox homeostasis [49], [50], [51], [52]. Interestingly, although GSH was reduced in the TRALI-like group compared to the Control group, it was higher than those observed in the LPS (i.p.) and LPS + saline groups. This may indicate that antioxidant components present in transfused plasma, such as exogenous GSH, partially buffered oxidative damage. A similar mechanism has been proposed in a previous report [53]. However, because the antioxidant composition of the transfused plasma was not directly quantified, this interpretation remains speculative and warrants future experimental validation.

Since MDA is a product of lipid peroxidation, which reflects the severity of cell damage caused by oxygen free radicals, and is closely related to ferroptosis dependent on lipid peroxidation [54], we further examined iron metabolism and lipid peroxidation. We found that iron concentration and MDA content were significantly higher in the TRALI-like group than in the Control group. However, it is important to note that elevated MDA and PTGS2 levels were also observed in the LPS (i.p.) group, indicating that LPS priming alone is sufficient to induce ferroptosis-associated oxidative and inflammatory signatures in lung tissue. These findings suggest that the ferroptosis-related biochemical alterations observed in the present study are not exclusive to the TRALI-like condition, but rather reflect a shared response to LPS-driven inflammatory and oxidative stress. Within this framework, plasma transfusion is more likely to modulate or amplify pre-existing LPS-induced ferroptotic susceptibility, rather than act as an independent initiating trigger. We hypothesise that oxidative stress and iron metabolism disorders under the TRALI-like condition may facilitate or exacerbate ferroptosis-associated pathways, thereby exacerbating lung tissue injury. Furthermore, the upregulation of PTGS2 may reflect the crosstalk between inflammatory signaling and lipid peroxidation–associated cell death processes, rather than serving as a TRALI-specific ferroptosis marker. Given its inflammatory responsiveness, PTGS2 cannot discriminate ferroptosis from generalized inflammation in this setting, and thus should be regarded as supportive rather than decisive evidence. However, the precise role and mechanisms of ferroptosis in TRALI remain unclear, particularly in the absence of direct ferroptosis inhibition or rescue experiments, which represents one of the limitations of this study. Overall, our findings indicate that ferroptosis, marked by iron overload, lipid peroxidation, and antioxidant depletion, may function as a downstream effector or amplifier of inflammatory lung injury in TRALI pathogenesis. This complements existing models of neutrophil-mediated inflammation and suggests that TRALI-related injury may be contextually amplified by ferroptotic processes [55]. Importantly, the present study primarily assessed downstream ferroptosis-associated biochemical changes and did not systematically evaluate upstream regulatory components of canonical ferroptosis pathways. Key regulators involved in lipid remodeling, antioxidant defense, and iron handling – including ACSL4, SLC7A11/xCT, FSP1, NRF2, and iron-regulatory proteins such as FTH1 or TFRC – were not examined. Consequently, the current data do not allow comprehensive pathway-level characterization of ferroptosis or definitive discrimination between ferroptosis and generalized oxidative injury. Future studies incorporating expanded ferroptosis marker panels, functional lipid ROS assays, and ferroptosis-targeted interventions will be required to refine mechanistic specificity and determine the causal contribution of ferroptosis to TRALI-like lung injury. From a mechanistic perspective, several upstream inflammatory and oxidative pathways may link TRALI-associated stress to ferroptosis activation. LPS priming and plasma transfusion can induce excessive reactive oxygen species production, potentially through Toll-like receptor 4 (TLR4) signaling, NF-κB activation, and mitochondrial dysfunction, which in turn disrupt iron homeostasis and lipid redox balance. Dysregulation of these pathways may create a permissive intracellular environment for ferroptosis activation in lung tissue. Although these pathways were not directly examined in the present study, they provide a plausible mechanistic framework connecting TRALI-related inflammation to ferroptosis and warrant further investigation. Because ferroptosis was not directly modulated or inhibited, the present data support an associative rather than causal relationship between ferroptosis-related changes and TRALI-like lung injury. Importantly, ferroptosis is pharmacologically targetable. Inhibitors such as ferrostatin-1 and liproxstatin-1 have demonstrated protective effects in models of ALI and sepsis. Identifying ferroptosis as a contributing mechanism in TRALI may therefore offer translational relevance, providing a potential therapeutic avenue beyond current supportive care approaches [44]. This study, to our knowledge, is among the first to link ferroptosis to TRALI, thus expanding the conceptual framework of transfusion-related lung injury. Future research should prioritize mechanistic validation using ferroptosis-targeted interventions and cell-type–specific approaches.

From a cellular perspective, ferroptosis may contribute to TRALI pathogenesis by modulating the function of multiple key cell types involved in the two-hit inflammatory cascade. Within the established two-hit framework, macrophages are increasingly recognized as central regulators of TRALI, particularly following the second hit, where they amplify inflammatory signaling and oxidative stress [56]. Ferroptosis-associated lipid peroxidation and iron dysregulation may alter macrophage redox homeostasis, thereby promoting macrophage activation or dysfunction and enhancing pro-inflammatory cytokine release, ultimately exacerbating alveolar epithelial injury [57]. Neutrophils represent another critical effector population in TRALI, and ferroptosis-related oxidative stress may further prime neutrophil activation, leading to increased production of reactive oxygen species, proteases, and neutrophil extracellular traps, which collectively contribute to lung tissue damage [40]. In addition, pulmonary microvascular endothelial cells play a pivotal role in maintaining alveolar–capillary barrier integrity, and ferroptosis-related disruption of iron homeostasis and antioxidant defenses may impair endothelial cell function, resulting in increased vascular permeability and pulmonary oedema [58]. Although cell-type–specific ferroptosis was not directly examined in the present study, integrating ferroptosis into macrophage–neutrophil–endothelium crosstalk provides a biologically plausible framework for understanding how iron-dependent lipid peroxidation may translate into TRALI-like lung injury and highlights important directions for future mechanistic investigations [59]. This conceptual framework is further supported by accumulating evidence from acute lung injury and sepsis-related models, which implicates ferroptosis in macrophage-driven inflammation, neutrophil-mediated oxidative injury, and endothelial dysfunction, thereby reinforcing the potential relevance of ferroptosis to TRALI pathogenesis [60].

Beyond mechanistic interpretation, the identification of ferroptosis-associated molecular alterations in this TRALI-like model provides important direction for future translational and data-driven research. The characteristic biomarker profile revealed in this study – including glutathione depletion, lipid peroxidation accumulation, iron dysregulation, and decreased GPX4 expression – represents a biologically coherent and mechanistically interpretable feature set, which may serve as a foundation for early diagnosis and risk stratification in transfusion-related lung injury. With the rapid development of artificial intelligence (AI) in biomedical research and clinical medicine, integrative analysis of multidimensional biological data has become increasingly feasible and clinically relevant. Recent advances, such as the AlphaFold model for protein structure prediction and the application of large language models for biomedical knowledge integration and clinical decision support, highlight the transformative potential of AI-driven approaches in medical research and healthcare [61], 62]. In this context, future studies could explore the incorporation of ferroptosis-related biomarker profiles, such as iron metabolism indicators and oxidative stress markers, into AI-based predictive models, with the aim of improving early identification of high-risk transfusion recipients and supporting individualized risk prediction strategies. Although such computational and AI-based approaches were beyond the scope of the present study, our findings provide an initial biological basis for the development of AI-assisted diagnostic and prognostic frameworks, thereby extending the significance of ferroptosis research in TRALI from mechanistic insight toward precision medicine-oriented applications.

This study has several limitations that should be acknowledged when interpreting the present findings. First, although the TRALI-like model employed here reproduced several key pathological and biochemical features of clinical TRALI, including pulmonary oedema and iron dysregulation, the absence of direct hypoxaemia measurements represents an important limitation with respect to fully recapitulating the physiological diagnostic criteria of clinical TRALI. According to established clinical definitions, acute hypoxaemia – typically defined as a PaO_2_/FiO_2_ ratio ≤300 mmHg or oxygen saturation (SpO_2_) <90 % – is a core diagnostic requirement for TRALI [63]. While pulmonary oedema is commonly assessed in animal models using wet-to-dry (W/D) weight ratios and histopathology, these parameters primarily reflect structural lung injury and cannot substitute for direct assessment of gas-exchange impairment, which is central to the clinical diagnosis of TRALI. Accordingly, the present model should be interpreted as a TRALI-like inflammatory lung injury model rather than a complete physiological equivalent of clinical TRALI. The lack of arterial blood gas analysis or oxygen saturation measurements therefore limits direct extrapolation to established clinical diagnostic standards [64]. In addition, although LPS (5 mg/kg) was used as a positive control to induce lung injury, the two-hit experimental design inherently introduces partial dependence on LPS priming, and thus the observed lung injury in the TRALI-like group may reflect an interaction between LPS-induced susceptibility and plasma-mediated amplification, rather than plasma-driven injury alone. Because a plasma-only transfusion group was not included, the independent contribution of plasma transfusion cannot be definitively isolated from the effects of LPS priming in the present study. Accordingly, future investigations should incorporate plasma-only transfusion controls, in parallel with saline volume replacement groups, to better delineate the relative contributions of LPS-driven inflammation and transfusion-related factors. Furthermore, the use of non-heat-inactivated human plasma in a rat model introduces the possibility of xenogeneic immune activation, including complement activation and other nonspecific inflammatory responses, which may partially mimic or exaggerate TRALI-like lung injury independently of classical TRALI mechanisms. Because the plasma used in this study was not heat-inactivated, we cannot exclude the contribution of complement-driven or other xenogeneic immune reactions to the observed pulmonary pathology. This represents an inherent limitation of cross-species transfusion models. To improve mechanistic specificity in future studies, additional plasma-processing control groups – such as heat-inactivated or leukoreduced plasma – should be incorporated to better distinguish xenogeneic immune effects from TRALI-related injury mechanisms within a two-hit framework.

Second, the lack of direct interventions targeting ferroptosis limits our ability to confirm its causal relationship with TRALI. Although our findings suggest an association between ferroptosis and TRALI, the conclusions of the present study were deliberately framed within an associative and hypothesis-generating context, and future studies using specific ferroptosis inhibitors or genetic models could provide more definitive evidence of causality. Additionally, although we observed key markers of ferroptosis (e.g. reduced GPX4 and GSH levels and increased iron and MDA content), these biochemical indicators alone are insufficient to definitively distinguish ferroptosis from generalized oxidative injury or LPS-driven inflammatory damage, and therefore should not be interpreted as direct evidence of ferroptosis-mediated causation. Consequently, direct modulation of ferroptosis would help further elucidate its role in the pathogenesis of TRALI. Moreover, ROS were not measured in this study, even though ROS have been shown to play a crucial role in neutrophil activation and TRALI development, as reported in previous studies [6], 33]. Although our results highlight oxidative stress markers such as lipid peroxidation, the absence of direct ROS quantification further constrains mechanistic interpretation and limits causal inference. Importantly, the Western blot analyses were performed with a limited number of biological replicates (n=3 per group), which substantially reduces statistical power and may have contributed to the absence of statistically significant differences between the LPS-only and TRALI-like groups. Therefore, the Western blot results should be interpreted as supportive rather than definitive evidence of group-specific mechanistic differences. Accordingly, future studies integrating ferroptosis-targeted interventions, ROS assessment, and increased Western blot sample sizes will be essential to determine whether ferroptosis plays a causal role in TRALI-like lung injury.

Furthermore, the choice of statistical analysis methods could impact the interpretation of results. The complexity of multi-group data requires more sophisticated statistical models to appropriately handle potential confounders. In the present study, one-way ANOVA was used as an appropriate method for initial group-level comparisons; however, future studies may consider more advanced statistical approaches such as multivariate analysis or longitudinal data analysis where appropriate to refine conclusions and improve the robustness of the results. Lastly, although lung histology in this study was qualitatively assessed, the inclusion of a standardised lung injury scoring system could provide a more objective and reproducible assessment of lung injury and inflammatory responses [65]. This would enhance the consistency and comparability of results across studies. Accordingly, the adoption of validated histopathological scoring criteria in future investigations is strongly recommended to strengthen pathological evaluation and reduce observer-dependent variability.

In addition, the plasma used in this study was derived from whole blood that was discarded from clinical transfusion. Consequently, detailed donor information was unavailable, and no testing for anti-leukocyte antibodies was conducted. This limitation precluded further stratification of antibody-mediated vs. non-antibody-mediated TRALI mechanisms, particularly with respect to distinguishing antibody-mediated and non-antibody-mediated pathways. Given that obtaining complete donor information is often impractical for discarded clinical plasma, future work will prioritise study-controlled, in-house plasma characterisation to improve interpretability, including screening for anti-HLA/HNA antibodies and targeted plasma component analyses, rather than relying primarily on donor metadata. Accordingly, future investigations should incorporate prospective plasma characterisation and component analyses to further improve mechanistic interpretability. Furthermore, we acknowledge a potential limitation related to the use of human plasma in a rat model. Previous studies have shown that cross-species transfusion can trigger nonspecific pulmonary responses due to complement activation or fibrinogen effects [65], 66]. Due to material limitations, only three animals per group were used for Western blot and image analysis. Although these data were intended to provide supportive molecular evidence, the limited sample size may affect the robustness of quantitative interpretation. Accordingly, future investigations should employ larger sample sizes, increased biological replicates, and standardized lung injury scoring systems to improve reproducibility and quantitative reliability.

## Conclusions

In conclusion, using a two-hit TRALI-like rat model (LPS priming followed by human plasma transfusion), we observed lung injury accompanied by a ferroptosis-associated biochemical and protein profile, including iron dysregulation, lipid peroxidation, GSH depletion, and reduced GPX4 expression. These alterations were also present across LPS-exposed groups, suggesting that they primarily reflect an inflammation-coupled oxidative stress response rather than a plasma-specific initiating mechanism. Human plasma transfusion may act as a second-hit amplifier of overall lung pathology on a primed background. Further studies incorporating plasma-only controls and ferroptosis-targeted interventions are warranted to determine the causal contribution of ferroptosis to transfusion-related lung injury.

## Supplementary Material

Supplementary Material
