# Supplementary material for: Ferroptosis-associated changes in transfusion-related acute lung injury in Sprague Dawley rats
Source: Open Med (Wars). 2026 May 11;21(1):20261418. doi: 10.1515/med-2026-1418 (PMC13157320; doi:10.1515/med-2026-1418)
Supplement: Supplementary file 1 — Supplementary Material [file j_med-2026-1418_suppl_001.docx]

**Supplementary Tables S1–S5**

**Supplementary Table S1. Raw data for lung wet/dry (W/D) ratio**

| **Group** | **Animal** | **Wet weight**  **(g)** | **Dry weight**  **(g)** | **Wet/Dry ratio** |
| --- | --- | --- | --- | --- |
| Control | 1 | 1.12 | 0.24 | 4.66667 |
|  | 2 | 0.91 | 0.18 | 5.05556 |
|  | 3 | 0.8 | 0.17 | 4.70588 |
|  | 4 | 0.73 | 0.15 | 4.86667 |
|  | 5 | 1.06 | 0.21 | 5.04762 |
| LPS (i.v., positive control) | 1 | 1.68 | 0.26 | 6.46154 |
|  | 2 | 1.54 | 0.24 | 6.41667 |
|  | 3 | 1.92 | 0.28 | 6.85714 |
|  | 4 | 1.44 | 0.22 | 6.54545 |
|  | 5 | 1.35 | 0.21 | 6.42857 |
| LPS (i.p., LPS-only) | 1 | 1.08 | 0.19 | 5.68421 |
|  | 2 | 1.14 | 0.21 | 5.42857 |
|  | 3 | 1.35 | 0.25 | 5.40000 |
|  | 4 | 1.26 | 0.19 | 6.63158 |
|  | 5 | 1.02 | 0.16 | 6.37500 |
| Saline (volume control) | 1 | 1.24 | 0.22 | 5.63636 |
|  | 2 | 0.93 | 0.17 | 5.47059 |
|  | 3 | 1.16 | 0.23 | 5.04348 |
|  | 4 | 1.31 | 0.21 | 6.23810 |
|  | 5 | 1.25 | 0.23 | 5.43478 |
| TRALI-like (LPS i.p. + human plasma) | 1 | 1.02 | 0.18 | 5.66667 |
|  | 2 | 0.98 | 0.17 | 5.76471 |
|  | 3 | 1.34 | 0.24 | 5.58333 |
|  | 4 | 1.28 | 0.23 | 5.56522 |
|  | 5 | 1.16 | 0.21 | 5.52381 |

Note: W/D ratio data; n = 5 rats/group. Group differences were tested by one-way ANOVA with Tukey post hoc comparisons. Exact P values, test statistics (with df), and effect sizes (with 95% CIs where applicable) are reported.

**Supplementary Table S2. Raw densitometry values for Western blot quantification (PTGS2 and GPX4)**

| **Group** | **Sample** | **PTGS2 intensity** | **GPX4 intensity** | **GAPDH intensity** | **PTGS2/GAPDH (calculated)** | **GPX4/GAPDH (calculated)** |
| --- | --- | --- | --- | --- | --- | --- |
| Control | 1 | 921 | 2563 | 2015 | 0.4571 | 1.272 |
|  | 2 | 1089 | 2268 | 1979 | 0.5503 | 1.146 |
|  | 3 | 1503 | 2117 | 2220 | 0.677 | 0.9536 |
| LPS (positive) | 1 | 2671 | 1032 | 2268 | 1.1777 | 0.455 |
|  | 2 | 2443 | 90 | 2198 | 1.1115 | 0.0409 |
|  | 3 | 2387 | 510 | 1935 | 1.2336 | 0.2636 |
| TRALI-like (experiment) | 1 | 2101 | 1428 | 2111 | 0.9953 | 0.6765 |
|  | 2 | 2006 | 1073 | 1962 | 1.0224 | 0.5469 |
|  | 3 | 1889 | 1930 | 2051 | 0.921 | 0.941 |

Note: Western blot densitometry (raw and/or normalized values as indicated); n = 3 rats/group. Analyses used one-way ANOVA with Tukey post hoc comparisons; exact P values, test statistics (with df), and effect sizes are reported. Due to the limited biological replicates, findings are supportive.

**Supplementary Table S3. Raw BCA protein concentration measurements for Western blot loading**

| **Group** | **Sample** | **Absorbance (mean)** | **Conc. (mg/mL)** | **Protein conc. (mg/mL)** |
| --- | --- | --- | --- | --- |
| Control | 1 | 0.8387 | 0.6786 | 6.7856 |
|  | 2 | 0.773 | 0.6255 | 6.2554 |
|  | 3 | 0.895 | 0.724 | 7.2403 |
| TRALI-like (experiment) | 1 | 1.1467 | 0.9272 | 9.272 |
|  | 2 | 0.9413 | 0.7614 | 7.6144 |
|  | 3 | 0.8263 | 0.6686 | 6.686 |
| LPS (positive) | 1 | 0.81 | 0.6554 | 6.5541 |
|  | 2 | 0.764 | 0.6183 | 6.1828 |
|  | 3 | 0.9637 | 0.7795 | 7.7947 |

Note: BCA-derived protein concentration values used for sample quantification/loading; no inferential statistics unless stated.

**Supplementary Table S4. Raw biochemical assay data (tissue iron, MDA, and GSH)**

| **Measure** | **Group** | **Sample** | **Value** |
| --- | --- | --- | --- |
| GSH (μmol/g protein) | Control | 1 | 29.4744 |
|  |  | 2 | 33.4436 |
|  |  | 3 | 30.99 |
|  | LPS (positive) | 1 | 9.2039 |
|  |  | 2 | 6.196 |
|  |  | 3 | 8.8879 |
|  | TRALI-like (experiment) | 1 | 15.3728 |
|  |  | 2 | 14.3091 |
|  |  | 3 | 18.3796 |
| Iron content (mg/g) | Control | 1 | 0.5636 |
|  |  | 2 | 0.4761 |
|  |  | 3 | 0.6917 |
|  | LPS (positive) | 1 | 1.5438 |
|  |  | 2 | 1.2917 |
|  |  | 3 | 1.1462 |
|  | TRALI-like (experiment) | 1 | 0.8832 |
|  |  | 2 | 0.8666 |
|  |  | 3 | 0.992 |
| Iron content (μmol/g protein) | Control | 1 | 10.0904 |
|  |  | 2 | 8.524 |
|  |  | 3 | 12.3857 |
|  | LPS (positive) | 1 | 27.6422 |
|  |  | 2 | 23.1284 |
|  |  | 3 | 20.5221 |
|  | TRALI-like (experiment) | 1 | 15.8146 |
|  |  | 2 | 15.5173 |
|  |  | 3 | 17.7626 |
| MDA (μmol/g protein) | Control | 1 | 0.9048 |
|  |  | 2 | 0.7729 |
|  |  | 3 | 0.8197 |
|  | LPS (positive) | 1 | 1.7838 |
|  |  | 2 | 1.655 |
|  |  | 3 | 1.5294 |
|  | TRALI-like (experiment) | 1 | 1.2443 |
|  |  | 2 | 0.9374 |
|  |  | 3 | 1.1364 |

Note: Biochemical indices (tissue iron, MDA, GSH); n = 5 rats/group (unless otherwise stated). One-way ANOVA with Tukey post hoc was used. Exact P values, test statistics (with df), and effect sizes (with 95% CIs where applicable) are reported.

**Supplementary Table S5. Summary of correlation analyses between ferroptosis-related indices and TRALI-like injury readouts**

| **Outcome** | **Predictor** | **Correlation (Pearson r)** | **P value** | **n** |
| --- | --- | --- | --- | --- |
| W/D ratio | GSH (μmol/g protein) | ≈ -0.923 | Not reported in provided file | Not specified (per file) |
|  | MDA (μmol/g protein) | ≈ 0.887 | Not reported in provided file | Not specified (per file) |
|  | Iron content | ≈ 0.92 | Not reported in provided file | Not specified (per file) |

Note: Correlation outputs based on the provided dataset. Correlation coefficient (r/ρ), exact P value, and n are reported (method specified in the table). Results are exploratory.
